# MicroRNAs in myocarditis: promising biological signals, but not yet a clinical test

**DOI:** 10.1016/j.ijcha.2026.101978

**Published:** 2026-07-27

**Authors:** Massimo Imazio

**Affiliations:** Department of Medicine, University of Udine, Udine, Italy Cardiothoracovascular Department, Santa Maria della Misericordia University Hospital, Udine, Italy

Myocarditis remains one of the most challenging syndromes in cardiovascular medicine. Its presentation ranges from an incidental increase in cardiac troponin to infarct-like chest pain, ventricular arrhythmias, advanced heart failure, cardiogenic shock, or sudden cardiac death. Moreover, myocarditis is not a single disease, but a heterogeneous inflammatory myocardial syndrome caused by infectious, immune-mediated, toxic, genetic, and occasionally overlapping mechanisms [1], [2], [3].

Current diagnostic pathways therefore rely on the integration of clinical presentation, electrocardiography, circulating biomarkers, echocardiography, cardiovascular magnetic resonance (CMR), coronary assessment when appropriate, and endomyocardial biopsy (EMB) in selected high-risk or diagnostically uncertain cases [1], [4], [5], [6]. None of these tools is perfect. Cardiac troponin identifies myocardial injury but is neither sufficiently sensitive nor specific for myocarditis. CMR can non-invasively demonstrate oedema and non-ischaemic myocardial injury, but its diagnostic performance depends on timing, protocols, disease phenotype, and local expertise. EMB provides unique histological, immunohistochemical, and molecular information, but its use is necessarily selective because it is invasive and affected by sampling error [1], [6].

Against this background, circulating microRNAs (miRNAs) are attractive biomarker candidates. These small non-coding RNA molecules regulate post-transcriptional gene expression and participate in inflammatory signalling, immune-cell differentiation, cardiomyocyte injury, fibrosis, and ventricular remodelling [7], [8]. Their relative stability in blood, particularly when transported within extracellular vesicles, offers the prospect of a biologically informative “liquid biopsy”.

In their systematic review and meta-analysis, Martinez Holst and colleagues examined 26 studies evaluating miRNAs in clinically diagnosed viral myocarditis [9]. They identified 42 differentially expressed miRNAs and reported an exploratory pooled diagnostic area under the curve (AUC) of 0.897, with pooled sensitivity of 0.758 and specificity of 0.881. The pooled AUC for discrimination of fulminant myocarditis was 0.883. Exosomal and circulating cell-free miRNAs showed broadly similar summary performance. These findings are encouraging, but their clinical meaning requires careful interpretation.

## 1 A biomarker class, not a single diagnostic test

The pooled AUC should not be understood as the performance of a defined test ready for clinical implementation. The meta-analysis combines different miRNAs, biological sources, analytical platforms, thresholds, patient populations, comparators, and diagnostic reference standards. In addition, multiple miRNAs estimates from individual studies were treated as statistically independent observations, potentially underestimating within-study correlation.

Consequently, the reported summary estimates describe the overall strength of the biological signal across the miRNA field rather than the accuracy of any one reproducible assay. This distinction is essential. A clinician cannot currently request “the miRNA test for myocarditis” with a validated threshold and a predictable likelihood ratio.

The high statistical heterogeneity reinforces this caution. I^2^ values exceeded 87% in the principal analyses, indicating marked between-study variation. Furthermore, many contributing studies were retrospective or case–control investigations comparing selected myocarditis cases with healthy controls. Such designs are useful for discovery but tend to produce more favourable diagnostic estimates than prospective studies involving consecutive patients with undifferentiated chest pain, elevated troponin, heart failure, or arrhythmias.

Publication bias is another concern. Positive findings involving novel biomarkers are more likely to be reported than negative or inconclusive experiments. The significant Egger test observed in the meta-analysis therefore raises the possibility that the available literature overestimates diagnostic performance.

## 2 What disease was actually diagnosed?

The term “viral myocarditis” also deserves scrutiny. In most contemporary practice, a recent viral syndrome does not establish viral myocardial infection. Viral material must be demonstrated within myocardial tissue using appropriately controlled molecular techniques, interpreted alongside histology and immunohistochemistry. Detection of circulating viral antibodies or a preceding respiratory illness is insufficient [1].

In the studies included in this meta-analysis, EMB and myocardial viral testing were inconsistently performed. Thus, the analysed population largely represents clinically suspected or imaging-supported myocarditis presumed to be viral, rather than uniformly biopsy-proven viral myocarditis. This introduces both disease misclassification and reference-standard bias.

This issue has practical implications for biomarker development. A miRNA released by injured cardiomyocytes may identify myocardial damage but not its cause. An inflammation-associated miRNA may distinguish myocarditis from healthy status yet perform less well against acute myocardial infarction, stress cardiomyopathy, genetic cardiomyopathy, systemic inflammatory disease, or immune checkpoint inhibitor-associated myocarditis. The clinically relevant question is not whether miRNAs differ between highly selected cases and healthy controls, but whether they add discriminative information in real-world diagnostic uncertainty.

## 3 Biological plausibility remains a major strength

Despite these limitations, several findings justify continued investigation. Some miRNAs appear linked not merely to myocardial necrosis but to specific inflammatory pathways. The proposed Th17-associated RNA hsa-Chr8:96, for example, showed promising discrimination in acute myocarditis and was evaluated against acute myocardial infarction rather than healthy controls alone [10]. Other candidates may capture different components of immune activation, cardiomyocyte injury, or remodelling [11], [12].

The future is therefore more likely to involve a multidimensional miRNA signature than a single marker. A panel combining inflammation-related and myocardium-enriched miRNAs might differentiate active myocardial inflammation from non-inflammatory injury and potentially identify distinct biological phenotypes. Integration with troponin, C-reactive protein, electrocardiographic findings, ventricular function, and CMR could prove more useful than direct competition with established tests.

However, incremental value must be formally demonstrated. A new biomarker should improve diagnosis, risk classification, or therapeutic decisions beyond information already provided by conventional assessment.

## 4 From association to clinical usefulness

Evidence supporting prognosis is even less mature. Fulminant myocarditis is a severe presentation identifiable at the time of evaluation, not in itself a conventional longitudinal outcome. Its discrimination should therefore be considered phenotypic classification rather than prognostic prediction. Associations between individual miRNAs and ventricular recovery, recurrent myocarditis, arrhythmias, transplantation, or death remain based on small and heterogeneous studies, sometimes with extremely wide confidence intervals.

Future studies should follow clearly characterised patients prospectively and distinguish three separate objectives: diagnosis of myocarditis, classification of a severe or mechanistically distinct phenotype, and prediction of subsequent clinical outcomes. Combining these objectives risks producing apparently impressive but clinically ambiguous results.

Standardisation is equally important. Serum, plasma, whole blood, peripheral blood mononuclear cells, myocardial tissue, and exosomes are not interchangeable matrices. Pre-analytical factors—including collection tubes, processing delay, centrifugation, storage, freeze–thaw cycles, haemolysis, and concurrent medication—can materially alter measured miRNA concentrations. Without harmonisation, even biologically valid findings may remain irreproducible.

## 5 Conclusions

This meta-analysis represents a valuable map of an evolving research field. It confirms that differential miRNA expression is repeatedly observed in myocarditis and identifies candidates worthy of focused validation. Nevertheless, the pooled estimates should be viewed as hypothesis-generating signals across a heterogeneous biomarker class, not as evidence for immediate clinical adoption.

The most productive next step is not another small study testing a different miRNA against healthy controls. What is needed is a prospective, multicentre diagnostic programme using standardised assays, clinically relevant comparator groups, rigorous diagnostic adjudication, and predefined validation cohorts.

MicroRNAs may eventually become part of a precision-medicine approach to myocarditis, complementing imaging, pathology, and conventional biomarkers. For now, they offer an exciting biological compass—but not yet a reliable clinical test (Figure).

**Table:**
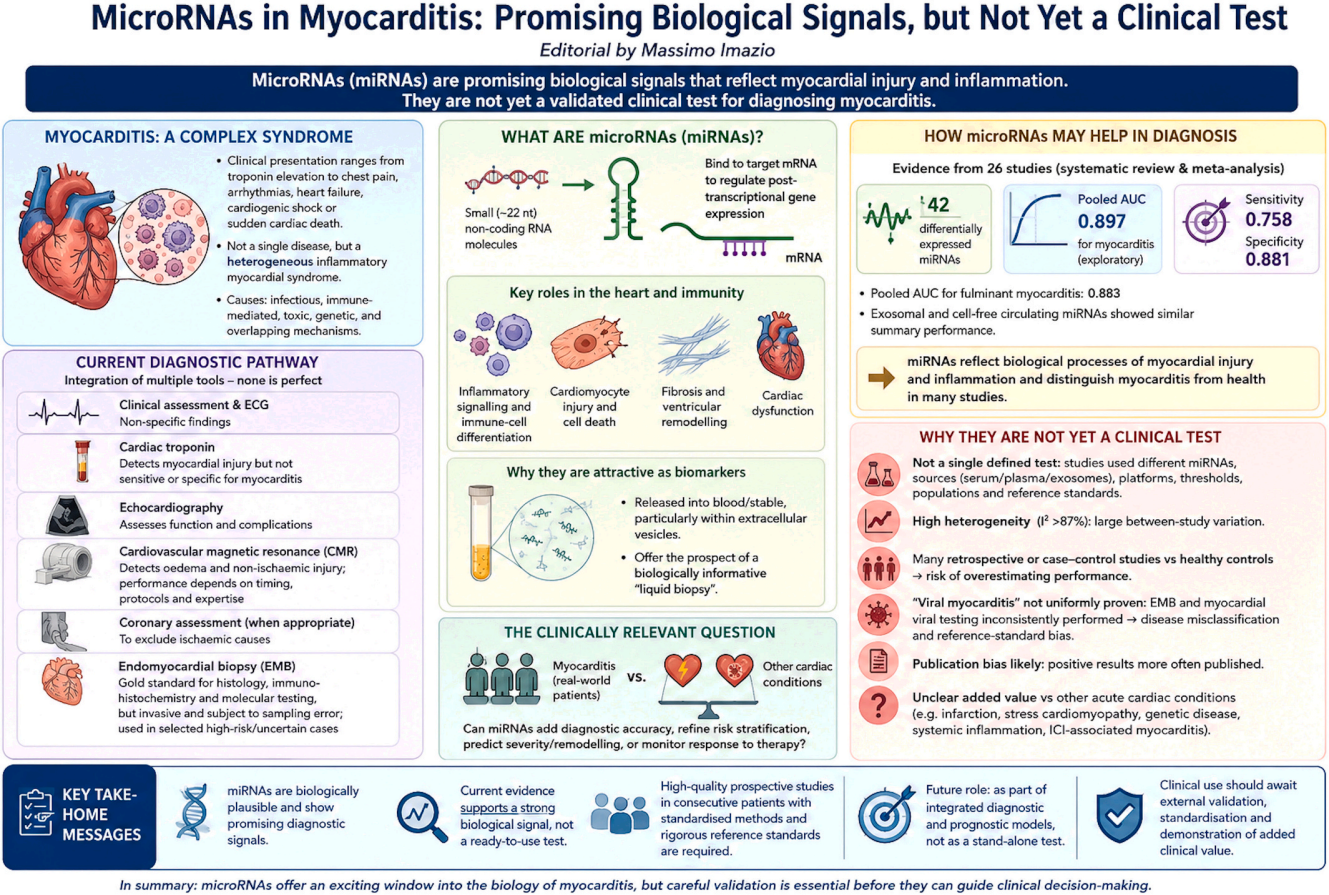


**Figure legend. MicroRNAs in myocarditis.** MicroRNAs are small non-coding RNAs involved in immune signalling, cardiomyocyte injury, fibrosis, and ventricular remodelling. Their stability in blood makes them promising candidates for a biologically informative ‘liquid biopsy’. Although current studies suggest good diagnostic discrimination, substantial methodological heterogeneity, inconsistent reference standards, and limited validation against clinically relevant comparators currently limit routine clinical use.

## CRediT authorship contribution statement

**Massimo Imazio:** Conceptualization, Data curation, Formal analysis, Investigation, Methodology, Supervision, Validation, Visualization, Writing – original draft, Writing – review & editing.

## Declaration of competing interest

The author declares that there are no conflicts of interest relevant to this work.
